# Emotional Eating Is Not What You Think It Is and Emotional Eating Scales Do Not Measure What You Think They Measure

**DOI:** 10.3389/fpsyg.2016.01932

**Published:** 2016-12-08

**Authors:** Peggy Bongers, Anita Jansen

**Affiliations:** Eating Disorders and Obesity, Clinical Psychological Science, Faculty of Psychology and Neuroscience, Maastricht UniversityMaastricht, Netherlands

**Keywords:** emotional eating, validity, self-report questionnaires, concerned eating, uncontrolled eating, cue-reactive eating

## Abstract

In eating research, it is common practice to group people into different eater types, such as emotional, external and restrained eaters. This categorization is generally based on scores on self-report questionnaires. However, recent studies have started to raise questions about the validity of such questionnaires. In the realm of emotional eating, a considerable number of studies, both in the lab and in naturalistic settings, fail to demonstrate increased food intake in emotional situations in self-described emotional eaters. The current paper provides a review of experimental and naturalistic studies investigating the relationships between self-reported emotional eater status, mood, and food consumption. It is concluded that emotional eating scales lack predictive and discriminative validity; they cannot be assumed to measure accurately what they intend to measure, namely increased food intake in response to negative emotions. The review is followed by a discussion of alternative interpretations of emotional eating scores that have been suggested in the past few years, i.e., concerned eating, uncontrolled eating, a tendency to attribute overeating to negative affect, and cue-reactive eating.

## Introduction

Although emotional eating was originally defined as eating in response to negative emotions, there currently are a number of studies that show that a positive mood can also elicit increased food intake (see for an overview Cardi et al., 2015). As such, several researchers have now accepted positive emotions as part of emotional eating. Eating in response to (negative) emotions can be problematic, as shown by studies that have related emotional eating to BMI (Laitinen et al., 2002; Konttinen et al., 2010; Péneau et al., 2013), weight gain (Hays and Roberts, 2008; Koenders and van Strien, 2011), interference with weight loss (Canetti et al., 2009), binge eating (Fischer et al., 2007; Ricca et al., 2009) and depression (Ouwens et al., 2009; Konttinen et al., 2010). In addition, emotional eating can have distressing immediate effects by for example leading to feelings of guilt (Wansink et al., 2003; Dubé et al., 2005; Macht and Dettmer, 2006). Although the concept of emotional eating may sound straight-forward, it is not as simple as is often assumed. In fact, the construct of emotional eating is more nuanced than is typically presented.

In order to study emotional eating, researchers should be able to measure it. For this purpose, several self-report questionnaires on emotional eating have been designed. The first of these, the Dutch Eating Behavior Questionnaire (DEBQ; van Strien et al., 1986), was developed in 1986. However, it was not until 13 years later that it was first incorporated in a study on the relationship between emotions and snacking (Conner et al., 1999). Since that day there has been a steady rise in studies on self-reported emotional eating, with the vast majority of those conducted from 2010 onwards. A greater interest in emotional eating within the scientific community has also seen an increase in self-report measures on the topic, with the development of the Emotional Eating Scale (EES; Arnow et al., 1995), the extended version of this scale (EES-II; Kenardy et al., 2003), the revised version of the Three Factor Eating Questionnaire—R18, (TFEQ-R18; Karlsson et al., 2000), the Emotional Appetite Questionnaire (EMAQ; Geliebter and Aversa, 2003), and the Emotional Overeating Questionnaire (EOQ; Masheb and Grilo, 2006). All these questionnaires are similar in design. They consist of a number of questions or statements regarding the desire for or frequency of food intake in response to negative—and sometimes positive—emotions, which are responded to on a Likert Scale, for example ranging from never to very often. Typical emotional eating items are: “Do you feel a desire to eat when you are feeling depressed or discouraged?” And “Do you feel a desire to eat when you are feeling lonely?” The underlying assumption of all questionnaires is that higher scores reflect a stronger tendency to (over) eat when experiencing (negative) emotions. Table 1 presents an overview of the questionnaire characteristics.

**Table 1:** **Characteristics of the different self-report emotional eating questionnaires**.

**Questionnaire**	**Items**	**Type of emotion**	**Example question**	**Likert scale**	**Subscales**
DEBQ—Emotional eating subscale (van Strien et al., 1986)	13	Negative	“Do you have a desire to eat when you are depressed or discouraged?”	1 (never) to 5 (very often)	Clearly labeled emotions (*n* = 9)
					Diffuse emotions (*n* = 4)
EES (Arnow et al., 1995)^a^	25	Negative	“Please indicate the extent to which the following feelings lead you to feel an urge to eat by checking the appropriate box.” This is followed by 25 feelings, such as “inadequate.”	1 (no desire to eat) to 5 (an overwhelming urge to eat)	Anger/frustration (*n* = 11) Anxiety (*n* = 9) Depression (*n* = 5)
EES-II (Kenardy et al., 2003)^a^	34	Negative Positive	“Please indicate the extent to which the following feelings lead you to feel an urge to eat by checking the appropriate box.” This is followed by 25 feelings, such as “angry” and “enthusiastic.”	1 (no desire to eat) to 5 (an overwhelming urge to eat)	Depression (*n* = 12) Positive mood (*n* = 11) Anger (*n* = 6) Anxiety (*n* = 5)
TFEQ-R18—Emotional eating subscale (Karlsson et al., 2000)^a^	3	Negative	“When I feel anxious, I find myself eating.”	1 (definitely false) to 4 (definitely true)	–
EMAQ (Geliebter and Aversa, 2003)^a^	22	Negative Positive	“As compared to usual, do you eat……” This is followed by 14 feelings and 8 situations, such as “when you are confident' and ‘after a heated argument.”	1 (much less) to 9 (much more)	Negative emotions (*n* = 9) Negative situations (*n* = 5) Positive emotions (*n* = 5) Positive situations (*n* = 3)
EOQ (Masheb and Grilo, 2006)^a^	6	Negative Positive	“On how many days out of the past 28 days have you eaten an unusually large amount of food, given the circumstances, in response to feelings of…” This is followed by 6 feelings, such as “happiness” and “sadness.”	0 (no days) to 6 (every day)	–

*DEBQ, Dutch Eating Behavior Questionnaire; EES, Emotional Eating Scale; TFEQ-R, Three Factor Eating Questionnaire—Revised; EMAQ, Emotional Appetite Questionnaire; EOQ, Emotional Overeating Questionnaire*.

a *The questionnaire is freely available*.

Interestingly, the nearly 20 years of research on self-reported emotional eating have had an unforeseen effect, namely to call into question the validity of self-report measures of emotional eating. Yet, self-reported emotional eating scales continue to be used and interpreted as simply being reflective of increased food intake in response to negative emotions, thereby ignoring numerous studies casting doubt on this claim. The aims of this paper are to raise awareness of (1) the issues that have arisen in the last decade with regard to emotional eating scales, and (2) alternative interpretations of high self-reported emotional eating scores that have been put forward. First, we present a literature review on studies that have examined the relationship between moods, food intake (both in the lab and in natural environments) and self-reported emotional eating. Second, based on the mixed findings in the literature, we go beyond what emotional eating questionnaires intend to measure, and present alternative interpretations of a high self-reported emotional eating score.

## Methods

A literature search was conducted using PsycINFO. Only articles written in English, and published in academic journals between 1986 (when the first self-report measure of emotional eating was introduced) and 2016, were considered. We started the search by looking specifically at titles, for which the following search terms were used (*n* = the number of articles for that search term): Emotion^*^ eat^*^ (*n* = 394); Stress^*^ eat^*^ (*n* = 193); Negative eat^*^ (*n* = 136); Mood^*^ eat^*^ (*n* = 107); Mood^*^ food (*n* = 57); Emotion^*^ food (*n* = 85); Stress^*^ food (*n* = 149); Negative food (*n* = 51). Subsequently we conducted a search within full articles for any mention of each of the self-report questionnaires: Dutch Eating Behavior Questionnaire (*n* = 220); Emotional Eating Scale (*n* = 56); Three Factor Eating Questionnaire (*n* = 135); Emotional Overeating Questionnaire (*n* = 4); Emotional Appetite Questionnaire (*n* = 1). Studies were included for review when they (1) measured self-reported emotional eating, (2) were conducted among adults 18 years and older, and (3) either had an experimental (mood induction and subsequent food intake in the lab) or naturalistic (assessment of mood states and food intake outside of the lab) design. Twenty-five studies met the criteria. Of these, 19 were experimental studies and 6 had a naturalistic design. With regard to the self-report measures, 20 used the DEBQ, 2 used the EES, 1 used the TFEQ-R, and 2 used other measures (i.e., a self-devised questionnaire). An overview of all studies is provided in Table 2.

**Table 2:** **Overview of experimental and naturalistic studies using self-reported emotional eating questionnaires**.

**Author**	**Design**	**Self-report questionnaire**	**Sample**	**Mood**	**Food**
Adriaanse et al., 2011—study 2	Naturalistic	DEBQ	Female students (*N* = 184)	Positive and negative emotions	Unhealthy snacks
Bongers et al., 2013a	Experiment	DEBQ	Male and female students (*N* = 87)	Negative: film excerpt Positive: film excerpt Neutral: film excerpt	Salted crisps, ketchup crisps, dark chocolate, milk chocolate, white chocolate
Bongers et al., 2013b	Experiment	DEBQ	Female students (*N* = 112)	Negative: film excerpt	Vanilla milkshakes
				Positive: film excerpt	
				Neutral: film excerpt	
Bongers et al., 2016	Experiment	DEBQ	Female students (*N* = 42)	Negative: memory recall (sadness) and sad music Positive: memory recall (happiness) and happy music Control: making puzzles	Six snack foods previously rated as highly palatable by the participant
Brogan and Hevey, 2013	Naturalistic	DEBQ	Obese adults (*N* = 57)	Positive and negative affect	Between-meal snacks, food eaten whilst cooking, all drinks, take-away meals, sugar added to drinks or meals
Conner et al., 1999	Naturalistic	DEBQ	Male and female students (*N* = 60)	Daily hassles	Food eaten between meals
Evers et al., 2009	Experiment	DEBQ	Female students		
Study 1			*N* = 30	Negative: emotional vignette	Chocolate, crisps, raisins, crackers
				Neutral: neutral vignette	
Study 2			*N* = 60	Negative: film excerpt Positive: film excerpt	Chocolate, crisps, raisins, crackers
Study 3			*N* = 37	Negative: memory recall (sadness)	Chocolate, crisps, cookies
				Neutral: memory recall (neutral)	
Study 4			*N* = 57	Negative: negative false feedback	Chocolate, crisps, fruit, crackers
				Positive: positive false feedback	
				Neutral: no feedback	
Fay and Finlayson, 2011	Experiment	DEBQ	Female students (*N* = 30)	Negative: memory recall (sadness)	Sweet popcorn
				Neutral: memory recall (neutral)	
Kenardy et al., 2003	Experiment	EES-II	Male and female students (*N* = 114)	Negative: task failure	Three types of savory crackers
				Positive: task success	
Kuijer and Boyce, 2012	Naturalistic	Other	Male and female adults (*N* = 105)	Natural disaster	Consumption of healthy amounts of food, fruit and vegetable intake, consumption of junk foods, overeating, eating breakfast
Newman et al., 2007	Naturalistic	DEBQ	Adult females (*N* = 50)	Daily hassles	Food eaten between meals
O'Connor et al., 2008	Naturalistic	DEBQ	Male and female adults (*N* = 422)	Daily hassles	Food eaten between meals
Oliver et al., 2000	Experiment	DEBQ	Male and female adults (*N* = 68)	Negative: anticipation of unprepared speech Neutral: listen to neutral text	Buffet consisting of fifteen bland, salty and sweet high fat and low fat foods
Raspopow et al., 2014	Experiment	DEBQ	Female students (*N* = 103)	Negative: anticipation of unprepared speech Neutral: read magazines	Miniature brownies
Royal and Kurtz, 2010	Experiment	DEBQ	Female students (*N* = 52)	Negative: unsolvable anagrams (high stress)	M&M's, Reese's Pieces, cheese crackers, peanuts
				Neutral: solvable anagrams (low stress)	
Schneider et al., 2012	Experiment	EES	Lean and obese adults (*N* = 60)	Negative: memory recall (anger/anxiety) Neutral: memory recall (neutral)	Six snack foods previously rated as highly palatable by the participant
Sproesser et al., 2013	Experiment	Other	Male and female students (*N* = 141)	Negative: social exclusion	Three types of ice cream
				Positive: social inclusion	
				Neutral: neither inclusion nor exclusion	
Turner et al., 2010	Experiment	TFEQ-R	Male and female students (*N* = 106)	Positive: film excerpt	Chocolate chip cookies
				Neutral: film excerpt	
van Strien et al. (2012—study 1)	Experiment	DEBQ	Female students (*N* = 124)	Negative: film excerpt Neutral: film excerpt	M&M's, crisps
van Strien et al. (2012—study 2/2013b/2014)^a^	Experiment	DEBQ	Female students (*N* = 47/47/54)	Negative: Trier Social Stress Test Neutral: Rating fabrics	Grapes, carrots, M&M's, butter cakes
van Strien et al., 2013a	Experiment	DEBQ	Female students (*N* = 60)	Negative: film excerpt Positive: film excerpt	Apple, banana, salty peanuts, sweet peanuts, crisps, jellies, cereal bar, chocolate, rice diet bar, rosquilleta
Wallis and Hetherington, 2004	Experiment	DEBQ	Females (*N* = 38)	Negative: ego-threatening Stroop task	Chocolate buttons
				Neutral: neutral Stroop task	
Wallis and Hetherington (2009—study 2)	Experiment	DEBQ	Females (*N* = 26)	Negative: ego-threatening Stroop task	Chocolate, dried fruit
				Neutral: neutral Stroop task	
Werthmann et al., 2014	Experiment	DEBQ	Female students (*N* = 85)	Negative: memory recall and negative music	Grapes, cucumber, chocolate, crisps
				Neutral: memory recall and neutral music	

a *These studies are grouped together because the same participant sample was analyzed in van Strien et al. (2012; study 2), van Strien et al. (2013b), and van Strien et al. (2014; including 7 additional participants)*.

## Literature review

### Experimental studies

The design of experimental studies on emotional eating generally consists of a mood manipulation (often sadness or stress, but other negative emotions have been evoked as well) followed by a bogus taste test in which participant's food intake is secretly measured. A few recent studies have also included positive emotions. The specific paradigms for mood manipulation vary over studies. Most commonly used are film clips and memory recall (sometimes in combination with mood-inducing music), followed by variations of the Trier Social Stress Task (TSST) and providing false feedback.

#### Film clips

In film paradigms, participants are instructed to watch a film clip (often an excerpt from a movie), most often selected to induce sadness. In one study, emotional eaters were found to eat significantly more when feeling sad compared to feeling joyful, while non-emotional eaters did not differ in their intake (van Strien et al., 2013a). In a comparable study, where sadness was compared to a neutral mood, emotional eaters increased their food intake when feeling sad, while non-emotional eaters decreased their intake (van Strien et al., 2012—study 1). However, closer inspection of the data revealed that emotional and non-emotional eaters consumed the exact same amount of food when sad, whereas there were large intake differences under neutral conditions. In contrast to these findings, Evers et al. (2009—study 2) found no differences in food intake between emotional and non-emotional eaters in either a negative or a positive mood. Likewise, in two other studies including negative, neutral and positive mood manipulations, there were no differences between groups when in a negative mood (Bongers et al., 2013a,b). However, in one of these studies, there was increased food intake in emotional eaters when they were in *positive* compared to a neutral mood (Bongers et al., 2013a). Finally, in a study comparing a positive to a neutral mood, no direct effect of self-reported emotional eating on cookie consumption was found (Turner et al., 2010). However, the researchers did find that the relationship between emotional eating and cookie consumption in a positive mood was mediated by uncontrolled eating (i.e., the ability to refrain from eating after being exposed to food cues).

Taken together, of the five studies inducing a negative mood, three fail to find any evidence for increased food intake in a negative mood in emotional eaters. One study does support the validity of emotional eater questionnaires, and one study's results are ambiguous. In addition, there is some evidence that positive mood can increase food intake in emotional eaters.

#### Memory recall

During memory recall procedures, participants are asked to remember a personally relevant emotional event, and instructed to either write it down or verbalize it. To create a stronger manipulation, memory recall can be accompanied by mood-inducing (personally chosen) music. The control procedure consists of recalling a neutral memory, such as the way to travel from home to work.

Schneider et al. (2012) showed a predictive effect of the anxiety subscale of the EES with regard to food intake following an anxiety induction, but the same was not found for the EES anger subscale in combination with an anger induction. In a study on food consumption while experiencing negative affect (Fay and Finlayson, 2011), a strong correlation between emotional eating score and intake was found. Additionally, after creating subgroups based on dietary restraint (i.e., restriction of food intake) and disinhibition (i.e., a combination of emotional eating and external eating, i.e., eating in response to external food cues, such as sight or smell of food) scores, the researchers reported significantly higher intake during negative affect in the high restraint/high disinhibition group compared to the low restraint/low disinhibition group. However, given the mixed characteristics of these groups, it is impossible to pinpoint the specific influence of emotional eating. In a final study that made use of recalling sad events, Evers et al. (2009—study 3) did not find any differences in intake between emotional or non-emotional eaters in either the negative or control condition. Two studies have combined memories with music. Werthmann et al. (2014) induced a negative or a neutral mood in their participants, but did not find an effect of self-reported emotional eating status on food intake. In a recent study in which memory recall was paired with personal music, Bongers et al. (2016) found a moderate correlation between self-reported emotional eating and food intake in both a negative and positive mood. In addition, they found increased food intake when feeling both negative and positive in emotional eaters compared to non-emotional eaters.

To conclude, there is some evidence that emotional eaters consume more food after recalling personal emotional events, although just as many studies cannot confirm these findings.

#### Trier social stress task

Variations of the Trier Social Stress Task are employed to induce stress. Participants are told that they will have to give a speech to an audience, while being judged and videotaped. They then receive a few minutes to prepare that speech. Sometimes, they are also informed that the speech will be followed by performing a difficult arithmetic task, which will also be judged and videotaped.

Van Strien and colleagues (same data set was analyzed and published in three different papers; van Strien et al., 2012—study 2; van Strien et al., 2013b, 2014) reported increased food intake in emotional eaters after performing the TSST compared to a control manipulation, while this was inversed for non-emotional eaters. Oliver et al. (2000) measured food intake during the time participants had to prepare their speech. They found no differences in total food intake or energy intake in stressed emotional eaters versus other groups, but did report that emotional eaters consumed more sweet high-fat foods and ate a more energy-dense meal. A final study employing the TSST found larger food intake in stressed emotional compared to stressed non-emotional eaters, although this failed to reach significance (Raspopow et al., 2014).

In sum, only one study found evidence for increased food intake in emotional eaters when stressed. In addition, one study reported a trend toward this effect, while a third study did not find differences in intake.

#### False feedback

Two studies manipulated mood by providing false (failure or success; performance compared to peers) feedback on task performance (Evers et al., 2009—study 4; Kenardy et al., 2003). Neither of these studies was able to demonstrate heightened food consumption in emotional eaters upon receiving either negative or positive feedback.

#### Other paradigms

A few studies have used less common methods to change mood. In a social exclusion paradigm, increased ice cream consumption was observed among students who reported they habitually eat more during stress compared to students who reported eating less (Sproesser et al., 2013). Emotional eaters did not differ from non-emotional eaters in their food intake after reading negative emotional vignettes, nor was there a difference in intake after reading negative compared to neutral vignettes (Evers et al., 2009—study 1). In a stress-induction study, participants underwent either an incongruent Stroop Task (cognitive stressor), an emotional Stroop Task (ego-threatening stressor) or a control Stroop Task (non-stressful procedure). Results showed increased chocolate consumption in emotional eaters after the ego-threatening stressor compared to the control condition, but no differences between the cognitive stressor and control (Wallis and Hetherington, 2004). However, in a second study, comparing the emotional and control Stroop tasks (Wallis and Hetherington, 2009), there was no effect of emotional eater status on intake. Stress has also been induced by giving participants either easy (non-stress condition) or unsolvable anagrams (stress condition) (Royal and Kurtz, 2010). Again, self-reported emotional eating did not affect food consumption.

All in all, one study found increased food intake in emotional eaters, one study found increased food intake only when a ego-threatening stressor as present, and three studies did not provide any support for increased food consumption in self-reported emotional eaters.

#### Summary

Experimental studies do not unequivocally support the assumption that high scores on self-reported emotional eating are predictive of actual increased food intake when feeling negative. Although some studies provide evidence in favor of this view, most research is either in contrast or shows ambiguous results. There is no consistent evidence for increased food intake under emotional circumstances in individuals scoring high on self-reported emotional eating.

### Naturalistic studies

To study emotional eating in a more natural environment, a number of naturalistic studies have been conducted. In these studies, participants are asked to fill out a diary in which they note their daily hassles and/or mood states, and food intake. Daily hassles refer to events, thoughts and situations that produce negative feelings (e.g., annoyance, worry) and interfere with goal achievement (O'Connor et al., 2008). As such, they can be considered a factor that triggers eating in emotional eaters.

#### Diary studies

Three studies investigated the effects of daily hassles on snacking (i.e., intake of food in between meals). Only one study found that snacking in response to daily hassles was moderated by self-reported emotional eating (O'Connor et al., 2008). In this study, participated recorded their daily hassles (and rated them on intensity) and food consumption in a diary for 4 weeks. Results showed that daily hassles (specifically those that were ego-threatening, interpersonal, or related to work) were positively related to consumption of high fat and high sugar foods, and that this relationship was moderated by emotional eating. In addition, the hassles-snacking relationship was also stronger for individuals higher in external eating and dietary restraint. Two similar studies that asked participants to fill out diaries on daily hassles and snacking did not report emotional eating scores to be of specific importance. Newman et al. (2007) found that the relationship between daily hassles and snacking was moderated by cortisol reactivity, in that higher cortisol reactors (i.e., individuals who produce more of the stress-hormone cortisol when stressed) snacked more in response to hassles while low cortisol reactors did not. In the high cortisol reactors, the relationship was stronger when levels of emotional eating were higher, but to the same degree as when individuals reported higher external eating and dietary restraint. Finally, Conner et al. (1999) demonstrated that external eating moderated the hassles-snacking association. They found no effect of emotional eating.

Two other studies focused on mood and affect, as opposed to daily hassles. Adriaanse et al. (2011) asked their participants to report mood and unhealthy snacking for 1 week, and found that the strength of habitual snacking and dietary restraint explained unhealthy snacking, whereas emotional eating did not. In a study on food intake in morbidly obese participants, emotional eating had no effect on overall food intake or intake of specific foods in either negative or positive mood (Brogan and Hevey, 2013).

In sum, one study found evidence for a moderating role of emotional eating in the relationship between daily hassles and snacking. One study reported a stronger relationship between hassles and snacking for those high on emotional eating, but found this for higher scores on external eating and dietary restraint as well. Three studies failed to find a role for emotional eating in the relationship between daily hassles or negative mood and snacking.

#### Natural disaster

Kuijer and Boyce (2012) conducted a study in the wake of a natural disaster. They assessed eating behavior in a community sample before and after the area of Christchurch, New Zealand, was struck by a major earthquake. Results showed that women who were self-reported emotional eaters reported an increase in overeating after the earthquake, but only when they also experienced high levels of earthquake-related distress.

### Methodological differences

One could argue that the lack of consistent findings is due to methodological differences. Across studies there is great variety in participant samples, mood inductions, and food available for consumption. One can reason that it might not be the case that self-report questionnaires are not valid, but rather that the mood induction paradigms or laboratory eating tests are the problem. It could be argued that none of the lab-paradigms equal the real-life experience of emotions, and that laboratory taste tests are very different from food consumption in real life. Although this is a valid point, similar mixed results have been obtained in naturalistic studies, which are capable of assessing real-life emotions and eating behavior. In addition, a recent study using Ecological Momentary Assessments (EMA—the repeated sampling of an individual's thoughts, feelings and behaviors in real time) did not find an association between self-reported emotional eating scores and food intake elicited by negative emotions (Boh et al., 2016).

Of note is that experimental studies that did find some evidence for the validity of emotional eating questionnaires generally included only extreme scorers (above the 80th and below the 20th percentile) (van Strien et al., 2012, 2013a,b, 2014). This could indicate that emotional eating might not be a very robust phenomenon and is not as ubiquitous as the media and general opinion lead us to believe. In addition, mood states that elicit overeating and the specific food that is preferably consumed when emotional can vary greatly among individuals. As such, self-reported emotional eating questionnaires might be valid, but only under very specific circumstances: when the self-reported score is extremely high, or when the exact right combination of mood state (e.g., sadness or anger) and available food (e.g., chocolate or crisps) for a certain individual is present. If so, an important question is what emotional eating questionnaires tell us in all other cases. If participants scoring high and low on emotional eating measures do not differ on intake when feeling negative, that is, if these questionnaires do not assess actual emotional eating, then what do they measure?

## What do high scores on emotional eating scales really reflect?

### Emotional eating scores in relation to other measures

Emotional eating has been positively related to BMI (Laitinen et al., 2002; Konttinen et al., 2010; Péneau et al., 2013) as well as a variety of behaviors and pathologies, including weight gain (Hays and Roberts, 2008; Koenders and van Strien, 2011), binge eating (Fischer et al., 2007; Ricca et al., 2009), depression (Fischer et al., 2007; Ouwens et al., 2009; Konttinen et al., 2010), self-reported impulsivity and lower inhibitory control (Ebneter et al., 2012; Jasinska et al., 2012) and several personality traits such as neuroticism, self-consciousness and lower self-sufficiency and self-esteem (van Strien et al., 1985; Heaven et al., 2001; Elfhag and Morey, 2008). However, if a high emotional eating score does not necessarily reflect increased food intake when feeling negative, as the foregoing literature review suggests, one cannot simply conclude that these studies have found an association between “increased food intake in a negative mood” on one hand and outcomes like depression, BMI or impulsivity on the other hand. Understanding the true meaning of a high self-reported emotional eating score is crucial for the interpretation of all studies on this topic.

### High scores might reflect lack of control, general eating concerns, a tendency to attribute overeating to negative affect, or learned cue reactivity

In recent years, some suggestions have been made regarding the interpretation of emotional eating scores. Vainik et al. (2015) studied questionnaires on five traits commonly considered to be related to eating, namely power of food (i.e., the psychological influence of the food environment), eating impulsivity (i.e., occasional loss of control over eating), disinhibition (i.e., tendency toward overeating), binge eating (i.e., eating a large amount in a short time while experiencing lack of control), and emotional eating. They concluded that all these traits share the same general underlying trait, which they labeled uncontrolled eating. More specifically, all five traits, including emotional eating, are thought to reflect a more general concept of low perceived self-control and high motivation to eat.

Two groups of researchers independently proposed “concerned eaters” as a more fitting description for emotional eaters (Adriaanse et al., 2011; Jansen et al., 2011). Adriaanse et al. (2011) found that emotional eating scores were predictive of a higher degree of worrying about one's eating behavior, a higher level of monitoring one's eating behavior, decreased perception of having control over one's eating behavior, and a higher extrinsic motivation to eat healthy. They suggest that emotional eating questionnaires might be more likely to measure the way individuals *think* about the relation between negative mood and eating, as opposed to actual food intake. In line with this, stressed emotional eaters have been found to overestimate their caloric intake compared to non-stressed emotional eaters, while not differing on actual caloric consumption (Royal and Kurtz, 2010). Jansen et al. (2011) based their suggestion for concerned eaters on a study that revealed that all DEBQ-identified types of eaters (i.e., emotional, external, restrained) showed the exact same pattern of food consumption after food cue exposure (i.e., smelling tasty foods without eating them). Based on these findings and a moderate-to-strong correlation between the emotional and external subscales, they argued that the DEBQ lacks discriminative validity and that higher scores on the scales are indicative of a general eating-concern.

A third plausible alternative interpretation of emotional eating scores was provided by Adriaanse et al. (2016), who argued that for some individuals, high emotional eating scores reflect a tendency to attribute past overeating to negative affect. Adriaanse et al. (2016) suggested that individuals cannot always explain their (overeating) behavior, and therefore might make up a reason (e.g., negative mood) that makes most sense to them. To test this assumption, participants were first asked to watch a short neutral video and subsequently eat exactly 20 grams of food. The next day, the researchers provided participants with false norm-violating feedback (i.e., that they ate substantially more than required) or control feedback. Participants were then given the opportunity to confabulate a reason for their overeating, by having to retrospectively rate their mood at the time of the estimation task. Results showed that individuals who scored high on emotional eating and had received the norm-violating feedback, retrospectively rated their emotions after the neutral film as significantly more negative than individuals with low emotional eating scores. In the control-feedback condition, high and low emotional eaters did not differ on their retrospective mood ratings. Thus, emotional eaters appeared to use “negative mood” as a confabulated reason for their overconsumption.

Finally, a recent study (Bongers et al., 2016), reasoned that emotional eaters could be better defined based on their actual food intake when in a negative mood: participants who ate the most after negative mood induction were considered emotional eaters. In counterbalanced order, the participants underwent a negative mood induction, a positive mood induction, food cue exposure, and a control procedure (all within subjects). Food intake was measured after each manipulation. It was found that emotional eaters consumed more food than non-emotional eaters, not only in the negative mood but in all conditions. If the participants were identified as emotional eaters based on their self-reported emotional eaters scores the same pattern was evident: high scorers on emotional eating ate significantly more than low scorers in all conditions. These findings are in line with a previous study which also found increased intake in emotional eaters after positive emotions (Bongers et al., 2013a), and with studies that demonstrate positive correlations between emotional and external eating (van Strien et al., 1986; Heaven et al., 2001; Ouwens et al., 2009; see for example Brignell et al., 2009; Turner et al., 2010; Jansen et al., 2011). The results suggest that emotional eating might be an indication of overeating in general, and not specifically in the presence of negative emotions. A variety of cues can become associated with eating—and therefore elicit learned cue reactivity—such as the sight and smell of food, the environment one is in, time of day, physiological states, cognitions and memories (Jansen et al., 2016). A term like “cue-reactive eaters” might therefore be more appropriate to describe the people whom we now call emotional eaters, and a distinction between emotional and external eaters would not be relevant.

To sum up, high scores on emotional eating scales are related to high scores on several other scales. It has been suggested that they reflect general eating concerns, lack of control, a tendency to attribute overeating to negative affect or being a cue reactive person. These four new interpretations of high scores on emotional eating scales all consider emotional eating to be different from mere and specific overeating in a negative mood. However, they differ in that concerned eating and a tendency to attribute overeating to negative affect are characterized by a continuous concern about and preoccupation with eating behavior that is cognitive in nature, whereas uncontrolled and cue-reactive eating are descriptive of actual eating behavior. How concerned eaters, a tendency to confabulate reasons for overeating, uncontrolled eating and cue-reactive eating are exactly related is a topic for future research.

## New ways to measure emotional eating

An alternative to the currently used explicit measures of emotional eating, which are susceptible to intentional and unintentional biases (Allison and Heshka, 1993; Adriaanse et al., 2011), is the use of implicit measures. In contrast to questionnaires which assess deliberate and conscious responses to the relationship between mood and eating, implicit measures should be able to assess more automatic and unconscious associations. One such measure, the IAT (Greenwald et al., 1998) is a computer task that implicitly assesses associations between two concepts. We developed a Single Target Implicit Association Test (ST-IAT) to assess associations between both negative emotions and eating and positive emotions and eating (Bongers et al., 2013b), reasoning that emotional eaters would have stronger associations between mood and eating than non-emotional eaters. The positive ST-IAT showed good predictive validity. Participants with stronger associations between positive emotions and eating consumed more food in a subsequent taste test in a positive mood. However, predictive validity was not observed for the negative ST-IAT. Participants with strong associations between negative emotions and food consumed more when in a positive mood but not when in negative mood. Considering the design of that particular ST-IAT, which used neutral words, emotional words, and food pictures, it is possible that instead of measuring actual eating behavior in positive or negative moods, it measured positive and negative feelings about high-energy palatable food. It might be interesting to redesign the ST-IAT to more accurately reflect eating (as opposed to food in general) and mood, and to validate it in real-life environments as well. Other implicit measures could also be of interest to identify emotional eaters. For example, approach and avoidance tendencies toward food (Brignell et al., 2009; Veenstra and de Jong, 2010; Havermans et al., 2011) or responses to a Food Stroop Task (Ben-Tovim et al., 1989) could be assessed in a negative or positive mood. It can be argued that emotional eaters would be faster to approach food and slower to name colors of food words when in a negative (or positive) compared to a neutral mood, while this would not differ in non-emotional eaters.

An explicit and promising new method is the use of Ecological Momentary Assessments (EMA), which involves repeatedly sampling a participant's thoughts, feelings and behaviors in real time. Through EMA, researchers can gain insight into the co-occurrence of specific feelings and food intake. One great advantage of EMA compared to self-report questionnaires is that EMA is not as susceptible to bias. Because emotions and food intake are assessed in real time, the problems with having to recall eating behavior, mood states, and their association are completely circumvented. In addition, individuals are not biased to think back of confirmatory evidence that is in line with the format of the questions (e.g., “do you have a desire to eat when you are depressed” might lead individuals to think of instances when this did occur, while paying less attention to instances when it did not) which could distort true emotional eating levels. Furthermore, eating behavior is measured in the moment in a natural environment, as opposed to a laboratory setting or being recorded at the end of the day in a diary. In recent years, EMA has been used repeatedly to assess relationships between mood states and food intake in both healthy and clinical populations. For example, several studies have investigated mood states in relation to binge eating in patients with Bulimia Nervosa or Binge Eating Disorder and find that negative moods precede eating binges (Wegner et al., 2002; Hilbert and Tuschen-Caffier, 2007; Smyth et al., 2007; Goldschmidt et al., 2014). In obese adolescents, daily hassles were reported to precede desires to eat (Kubiak et al., 2008). Studies in overweight and obese dieters have shown that both positive and negative moods are related to dieting lapses (i.e., breaking the diet) and experiencing temptation to eat (Carels et al., 2002, 2004). Two other studies reported a larger role for positive compared to negative emotions in eliciting eating behavior in both healthy and overweight adults (Macht et al., 2004; Boh et al., 2016).

When using EMA to assess whether someone is an emotional eater, one could compare the occurrence of negative emotions followed by eating relative to the occurrence of negative emotions without subsequent food intake. The greater the proportion of negative emotions followed by food consumption, the more likely someone is to be an emotional eater. With regard to the alternative interpretations of emotional eating, cognitions can be measured through EMA to link eating concerns to emotional eating behavior. Likewise, EMA-assessments of eating in response to negative emotions can be linked to eating in response to for example positive emotions and external food cues to investigate the ideas of uncontrolled and cue-reactive eating.

Finally, although there appear to be viable alternatives to self-report questionnaires when it comes to identifying emotional eaters, we are not likely to eschew questionnaires entirely in the near future. Implicit measures of emotional eating are in need of validation (for example by linking them to EMA outcomes), while EMA can be too expensive and time-consuming when a quick assessment of emotional eating is required. Therefore, it is worthwhile to consider ways to improve the validity of self-report questionnaires. One characteristic of current questionnaires that could induce bias is that they are always framed in a positive way (i.e., “do you have a desire to eat when you are depressed” or “when I feel anxious, I find myself eating”) which might lead individuals to think of instances in which they indeed felt depressed or anxious and ate, while ignoring all other instances in which they felt that way but did not eat. Reframing of the questions so that part of the questionnaire needs to be reverse scored (i.e., “do you have a decreased desire to eat when you are depressed” or “when I feel anxious, I find myself eating less”) could reduce the bias in responding. Another possibility could be to instruct individuals to think of a specific time period, i.e., the past 2 weeks, to prevent recollection of emotional eating events that happened a long time ago and are given undue significance.

## Conclusion

In this paper we have raised two issues with regard to emotional eating. We have (1) questioned the validity of self-report emotional eating questionnaires, and have (2) discussed alternative interpretations of these measures. The assumption that emotional eating questionnaires are adequate measures of eating behavior in response to negative emotions is no longer tenable, as shown by the abundance of studies demonstrating no increased food intake in negative moods in self-reported emotional eaters. Together with the moderate to strong correlations between emotional eating and other indices of overeating or eating concerns, these results suggest that the concept of emotional eating is more complicated than it is often thought to be. Although the exact nature of emotional eating remains elusive, it is clear that current questionnaires cannot be relied upon to measure this behavior, and there likely is more to emotional eating than increasing food intake specifically when in a negative mood. Researchers interpreting findings based on self-report emotional eating measures, be it in their own data or in results of others, should be careful and critical when doing so.

## Author contributions

Substantial contributions to the conception or design of the work (PB, AJ). Drafting the work (PB) or revising it critically for important intellectual content (PB, AJ). Final approval of the version to be published (PB, AJ). Agreement to be accountable for all aspects of the work in ensuring that questions related to the accuracy or integrity of any part of the work are appropriately investigated and resolved (PB, AJ).

## Funding

This review is part of an ongoing project that is financed by the Netherlands Organisation for Scientific Research (NWO): Vici Grant 453.10.006, awarded to AJ.

### Conflict of interest statement

The authors declare that the research was conducted in the absence of any commercial or financial relationships that could be construed as a potential conflict of interest.
